# How Climate Change Science Is Reflected in People’s Minds. A Cross-Country Study on People’s Perceptions of Climate Change

**DOI:** 10.3390/ijerph19074280

**Published:** 2022-04-02

**Authors:** Ruxandra Malina Petrescu-Mag, Philippe Burny, Ioan Banatean-Dunea, Dacinia Crina Petrescu

**Affiliations:** 1 Department of Environmental Science, Faculty of Environmental Science and Engineering, Babeș-Bolyai University, 30 Fantanele Street, 400294 Cluj-Napoca, Romania; malina.petrescu@ubbcluj.ro; 2 Department of Economy and Rural Development, Faculty of Gembloux Agro-Bio Tech, University of Liège, Passage des Déportés 2, 5030 Gembloux, Belgium; philippe.burny@uliege.be; 3 Research Institute for Sustainability and Disaster Management Based on High Performance Computing (ISUMADECIP), Babes-Bolyai University, 30 Fantanele Street, 400294 Cluj-Napoca, Romania; 4 Faculty of Agriculture, Banat’s University of Agricultural Sciences and Veterinary Medicine “King Michael I of Romania”, 119 Calea Aradului Street, 300645 Timisoara, Romania; ioan_banatean@usab-tm.ro; 5 Department of Hospitality Services, Faculty of Business, Babeș-Bolyai University, 7 Horea Street, 400174 Cluj-Napoca, Romania

**Keywords:** adaptation, awareness, causes, citizens’ views, conflict, denialism, maladaptation, media

## Abstract

The way people perceive climate change scientific evidence becomes relevant in motivating or demotivating their climate actions. Climate change is one of the most publicized topics globally, and media has become an important “validator” of science. Therefore, science has become more exposed to criticism. Even when most scientists, decision makers, and laypeople agree on the robust evidence of climate science, there is still room for disagreement. The main aim of this paper is to reveal how climate change knowledge generated by science is perceived by the laypeople and to observe a possible gap between them. The study answered two questions “What are the main contrasting climate change topics in the scientific literature?” and “What are Romanian and Belgian participants’ perceptions of these topics?”. A qualitative approach was chosen for data analysis, using Quirkos software. The present cross-country study showed commonalities and differences of views between the two groups of participants regarding six climate change topics. Divergent perceptions among Belgians and Romanians came out, for example, within the theme “The heroes, villains, and victims of climate change.” Thus, whereas Belgians considered all people, including themselves, responsible for climate change, Romanians blamed mostly others, such as big companies, governments, and consumers. Additionally, both groups stated that climate change existed, but contrary to Belgians, Romanians voiced that climate change was often used as an exaggerated and politicized topic. The analysis revealed that perceptions about climate change, its causes, and its impacts are social constructs with a high degree of variability between and within the two national groups. The study argued that the cleavages between scientific literature and people’s views were blind spots on which a participatory approach was needed to better cope with climate change challenges.

## 1. Introduction

Climate change is one of the most significant challenges where economic, social, and political stakes are high. Climate change has moved from being mainly a physical phenomenon to being simultaneously a social [1], economic, and political one, and it has a plethora of interpretations and prognostics [2]. Even when most scientists, decision makers, and laypeople agree on the robust evidence of climate science, there is still room for disagreement, especially when scientific interpretations are not beyond doubt [3].

The public draws most of its knowledge about climate science from the media, considered a primary source of information [4]. Climate change is one of the most publicized topics globally. Media, which is often the interface between scientists and citizens, succeeds in powerfully influencing people’s awareness and public debates, with further implications in how governmental actions towards climate change mitigation are accepted and implemented [5]. Thus, it is justifiable to say that media has become an important “validator” of science [6], and consequently, science has become more exposed to criticism. Many times, science in general, and climate science in particular, have not been merely mirrored in the media but also “reconstructed” [2] with further impact on how media consumers react to it. The reception of science-based climate change evidence influences how people think and act towards climate change. At the same time, research has shown that personal experience is an essential determinant of climate change adaptation [7,8], and climate change narratives reflected in media, rather than climate science, are relevant in motivating or demotivating climate action [9,10,11]. Climate change narratives are valued as storylines about the climate crisis that embed the problems, causes, possible solutions, and even moral responsibilities [10,12]. They are articulated in many ways, and they are often situated within a wide range of environmental stories [3]. However, there is little consensus on an exact definition of a narrative [13].

Familiar climate narratives have become prominent through media, international organization conferences, reports, and scientific literature that have legitimized climate change narratives [14,15]. Therefore, the use of storylines both as an instrument for significant changes [16] and as a communication and engagement device is widely acknowledged [17]. The factual occurrences in a place and people’s values are connected through storylines that contribute to how knowledge is generated and communicated [18]. Narratives integrate particular events and trends in the worldviews of people who experience them [16]. Therefore, they are rooted in a socio-cultural context and act as a social structure [18,19]. The different interpretations of climate change storylines and contrasting reported climate change science evidence make us think that climate change is a “tower of Babel” realm [1,20]. Paul Matthews [21] showed that around 60 of 154 climate skeptics based their attitude on invoking that climate science is insufficiently rigorous. Public confusion is reflected by the existence of a diversity of opinions and behaviors regarding the urgency to approach climate change as a humanity problem [22,23].

We advanced the following exploratory questions (EQs) within this context: (1) what are the main contrasting climate change topics in the scientific literature? (EQ1) and (2) what are Romanian and Belgian participants’ perceptions of these topics? (EQ2). Three main objectives are set to respond to these EQs. The first objective is to identify contrasting scientific arguments about climate change in the literature. The second objective is to reveal how Romanian and Belgian participants think and feel about selected science-based climate change topics. The third objective is to identify the climate change portrayals that participants express for each science-based climate change topic. Practically, the main aim of the paper is to reveal how climate knowledge generated by science is perceived by laypeople and to observe a possible gap between them.

## 2. Materials and Methods

First, based on desk research, different aspects (mainly controversial, e.g., origin, impacts) related to climate change were identified and described based on the scientific literature and international documents. Six science-based climate change topics were identified. The search for the scientific literature was conducted in electronic databases (e.g., Scopus-Elsevier, Wiley Journals, Cambridge Journals, SpringerLink Journals, Springer, Web of Science-Core Collection, Emerald Management Journals 200, ScienceDirect Freedom Collection-Elsevier) downloaded from Anelis plus platform (Enformation portal). The search was restricted to papers written in English. The reference time frame was 1990 (publication date of the first IPCC Assessment Report)—2022. For 1990–2022, only one keyword—“climate change sources”—generated 41,456 records (journal papers, conference proceedings, books, and books series) in Scopus Elsevier (which is the largest abstract and citation database of scientific literature according to Schotten et al. [24]). Considering the vast number of published materials, the authors decided to analyze a maximum of 500 publications. In the next step, we discriminated between publications based on their titles, using a long list with searching keywords (e.g., “climate change sources”, “climate change origins”, “climate change denial”, “climate change skeptics”, “climate change controversies”, “climate change adaptation”, “climate change impacts”, “climate change solutions”, “maladaptation”, “climate change mitigation”, “IPCC”, “narratives”, “climate change narratives”, “climate change perceptions”, “climate change behavior”, “climate change attitude”). Once a paper was selected based on the title, the abstract was read in the following step, and 281 abstracts were retained. For these papers, we looked for their full text, and we performed a content analysis to retain the most relevant ones. For some of those retained, we checked Google Scholar for the number of citations and citing articles. Thus, new papers were identified within the “forward snowballing” phase. Finally, 105 full-text records were retained and analyzed.

The following science-based climate change topics were identified following this methodological approach: (1) “Climate change between awareness and denialism”, (2) “Causes of climate change”, (3) “Manifestations and solutions of climate change”, (4) “Climate change adaptation and maladaptation”, (5) “Climate change: a source of conflict”, and (6) “The heroes, villains, and victims of climate change”; here, (6.1) “The “King” of climate change villains” was highlighted. For subtopic 6.1, four main contributors to climate change (burning fossil fuels for electricity, transportation, industry, and households; changes in agriculture and land use, such as deforestation; waste landfills; use of fluorinated gases (F-gases)) were selected from The European Environment Agency [25] report. “Overproduction-overconsumption nexus” was selected based on Jones’ study [26], and the authors added “overpopulation.”

The second methodological step was to conduct a qualitative interview to reveal how the selected science-based climate change topics are locally embedded. The understanding about how science-based knowledge regarding climate change is perceived by laypeople is limited and, in this context, authors considered appropriate to adopt an exploratory approach. They opted for the qualitative research because it helps to gain an understanding of concepts, thoughts, and experiences, while quantitative research confirms or tests a theory or hypothesis. Qualitative research enables to gather in-depth insights into the problem or help to develop hypotheses for future quantitative research. By using the qualitative interviews to explore the beliefs and values regarding the selected science-based climate change topics, a human dimension was added to scientific evidence of climate change. Between November 2021 and February 2022, 29 qualitative face-to-face interviews that offered great discussion flexibility were collected (14 from Romania and 15 from Belgium). The focus on Romanian and Belgian participants reflects the need to offer a comparative image of how people from a former communist country (Romania) and an old free-market country (Belgium) perceive various aspects related to climate change. They are both EU member states but different in terms of geographical, economic, and social characteristics. Consistent with other research [27], we considered that the historical background that affected the politics, economics, social organization, and mentality of individuals, can influence their perspective on climate change. Additionally, it was more convenient to investigate people from these two countries because researchers know the language and the existing sociocultural, economic, environmental, and political context.

The semi-structured interviews generated a picture of participants’ perceptions of the six science-based climate change topics. The interviews were carried out in Romanian and French, respectively. They lasted between 35 and 45 min. The interviewers were two of the authors, one for Romanian participants and one for Belgian participants. They were trained by one of the authors with higher expertise in qualitative interviews. Interviewers’ training ensured no unintended influence on what the subject answered. The sampling strategy envisaged selecting participants from different groups (age, level of education, income and living area—rural and urban). We started from an initial group (people with whom researchers interacted/worked) for the recruitment of participants. Then, following the snowballing technique (meaning that a participant recommended another one who was further selected if she/he met the pre-established selection criteria), we reached the final number of participants.

The participation was voluntary, the confidentiality and anonymity were ensured, and no financial reward was given for participation. The participants were explained the aim of the study, why they were selected, and what would happen with the interview data. A filter question, “Have you heard about climate change?” was included. Nobody answered “no”; therefore, nobody was excluded from the interview. The semi-structured interviews asked questions that were planned and open-ended. More precisely, the interviewers had a list with broad questions for each science-based climate change topic, and they allowed flexible answers and discussions. To obtain a fuller image of what people think about each of the six topics, the interviewer was given a set of additional set questions (“interview guide”) to guide them within the interview process. This “interview guide” comprised core questions and many associated questions related to the central question [28] that were skewed towards the interests of the interviewer (examples of related questions: “Is climate change used as a pretext by politicians and scientists to manipulate us, to impose restrictions on us?”, “Do you think that it is an exaggeration to talk about climate change and its effects?”, “Do you think that climate change is already present in your region?” “Do you believe climate change is visible mainly in other parts of the world than your country/region?” “Especially on which continents/countries/regions do you consider climate changes impacts are present?”, etc.; the interviewer offered an extra explanation for the theme of Climate change adaptation and maladaptation), (Supplemental File S1).

At the 14th interview, 15th, respectively, new meanings and statements could no longer be revealed. Therefore, “theoretical saturation” [29] was reached, meaning that we had enough interviews.

The interviews were recorded and transcribed verbatim. In the transcripts, nicknames were assigned to respondents to facilitate the analysis. Finally, data input and analysis were carried out using Quirkos software version 2.4.1. A thematic analysis was performed. All six science-based climate change topics were coded (they were called “thematic codes”, “themes”, or “Quirks”). For the most cases, participants’ statements were not assigned directly to the “Quirks” since each “Quirk” was broken down into smaller meaningful units of themes called, from now on subtopics (e.g., “Awareness”, “Denialism,” “Exaggerations”, etc.). However, there were cases where people’s opinions could not be included in a separate subtopic. Those statements were attached directly to the “Quirk”, as it was the case for Quirk 4 and 5 (Themes 4 and 5). Therefore, those answers were included in the Quirk and not in a subtopic. Similarly, in Quirk 4 (Theme 4), we included the statements saying that people did not hear about successful programs/strategies/actions aimed at climate change adaptation. The authors opted to use Quirkos because is largely used for reporting qualitative data. It helps to read the data more efficiently. Because of the visual interface, it gives you the possibility to compare the results very easily.

## 3. The Science-Based Climate Change Topics: A Literature Review

This section presents climate change as a “wicked” multidimensional problem [30,31,32]. This term depicts the dynamics of the change with causes and effects at multiple scales of time, with diverse human-environmental interactions and conflicting inputs, and multiple possible outcomes [31] and solutions that create a “plethora” of new other problems [33]. The six selected climate change topics reflect this “wicked” multidimensionality of climate change. From the beginning, we emphasized that even if all six science-based climate change topics are interconnected, we wanted to individualize each topic as the “nucleus” of the selected scientific papers, around which the other topics are agglutinated. This section, practically, aims to answer the first EQ, “What are the main contrasting climate change topics in the scientific literature?” (see Table 1).

### 3.1. Climate Change between Awareness and Denialism

Conspiracy-based narratives, religious beliefs, personal attitudes and experiences of previous exaggerated scares, and even poor science are used to rationalize the climate change skepticism [21,43]. Huber [140] highlights the importance of populism in explaining individuals’ attitudes towards climate change politics, showing that populist attitudes are associated with climate skepticism. That is why, according to Rahmstorf [50], climate change denialism is reflected in various forms, such as the denial of and doubt about the climate change existence (trend skepticism), the anthropogenic causes as the main contributors of it (attribution skepticism), and the severe consequences of climate change (impact skepticism). The doubt on those producing scientific evidence (consensus skepticism) [41] or the inefficiency of legislative and political measures are also part of climate change denialism (political distrust) [38]. That is why we can affirm, once again, that all the selected science-based climate change topics are interweaving, and they are interdependent, and strict boundaries cannot be drawn between them. However, climate change denialism is primarily focused on two directions: one that denies the anthropogenic causes of climate change and another that downplays the extent to which climate change would bring about negative consequences [35,53].

Following Bonds [36], the climate-denialist movement is not an inconsiderable network, and the fossil fuel industry representatives, individual scientists, experts, or politicians are part of it. Moreover, Cann and Raymondwarns [37] warn that since the political debate evolves, the opposition to climate change takes new forms, with strong international reverberations. Climate change denialism research focuses mainly on the US, where climate skeptics are very vocal and present in mainstream debates [141,142]. According to Dunlop and Jacques [40], this is due to well-funded climate change denial. Poortinga et al. [48] report an increasing climate change skepticism in the US and Europe. The percentage of European citizens considering climate change as one of the most severe problems declined from 65% in 2008 to 43% in 2017 [39]. Despite this decline, the 2021 Eurobarometer on climate change informed that 93% of the surveyed people considered climate change a serious problem, and 78% of them as very serious [42]. In the former communist countries, people were less inclined to consider climate change as the single most serious problem facing the world, and there, the seriousness of climate change was perceived as lower than the EU average [42]. In Romania, for example, only 66% of people thought that climate change was a severe problem, a lower percentage than the EU average (78%), while in Belgium, almost 82% considered climate change a tough issue [42].

Studies on public perceptions of climate change are numerous (e.g., [45,49,51,52]), and they enhanced the understanding of how people feel and think about climate change. However, the levels of climate change awareness, knowledge, perceived risk, and support for mitigation or adaptation are country- and culture-specific, making generalization challenging [44,49].

### 3.2. The Causes of Climate Change

As the “devil is in the uncertainty” [65], this science-based climate change topic reflects upon the causes of climate change reported in the climate literature—anthropogenic, natural, and even climate change as “acts of God.”

A persistent question in the mainstream debate on climate change in general, and in global-warming in particular, refers to the extent to which anthropogenic causes cause them, or they are a manifestation of natural climate variability [58,60]. The latest IPCC report [62] states that human activities have unequivocally caused greenhouse gas emissions since around 1750. Human influence has warmed the climate at an unprecedented rate in the last 2000 years. Anderegg et al. [55] compiled a database of 1372 climate researchers, and they showed that around 98% of climate researchers support human-induced climate change causes outlined by the IPCC over time. Cook et al. [57], who researched the evolution of the scientific consensus on anthropogenic global warming in the scientific literature, examining 11,944 climate abstracts from 1991–2011, found that among the abstracts that reported a position on anthropogenic global warming, 97.1% stressed out that humans were causing global warming. Recently, Lynas et al. [46] examined 3000 papers published since 2012. They concluded on a scientific consensus on human-caused recent climate change (99% of the peer-reviewed papers pointed to this conclusion).

Crowley [59] comes with two conclusions—that the warming over the past century is unprecedented in the past 1000 years and that the climate model that can explain much of the variability in Northern Hemisphere temperature shows that only about 25% of the 20th-century temperature increase can be attributed to natural variability.

Following Mahlman [63], it is clear that human-caused greenhouse warming is not a problem that can be ignored. Still, the prediction of human-induced climate change requires models that can accurately simulate natural circulation regimes and their associated variability [58]. In addition, there are uncertainties in modeling important aspects of climate change that make a clear picture of how the warmed climate will proceed cannot be produced [63,65]. Climatic records show that widespread and impactful climate changes occurred throughout the geological record [54], but human-induced causes could cause them to happen again [65].

A way of framing climate change has been through a putative relationship between the laws of God and the laws of nature [61], climate change being judged as the consequences of human actions that have angered God [56]. Religious framings of climate change have been reported in various papers that have investigated people’s perceptions of climate change [64,66,67]. This proves that the battles over climate change occur both in a cultural environment and in atmospheric spaces [61].

### 3.3. Manifestations and Solutions as Disconnected Narratives of Climate Change

This section presents two main aspects of climate change: one about the manifestations of climate change and the other about the solutions. We start from Randall’s [80] paper, where the loss (e.g., biodiversity loss, loss of habitat, water scarcity, food shortage, loss of livelihood) features dramatically but is located in the future or in places remote from western societies; when it comes to solutions, the loss is most often left out of the narrative. However, when we refer to food waste mitigation practices, for example, we can also have losses, shortages, and deficiency of food. We are part of a culture in which an enormously productive economy demands that consumption becomes a way of life, with the purchase and use of goods transforms into rituals [143]. Our food and dietary choices impact climate change, since the emissions of the major gases that contribute to the greenhouse effect are related with food production and consumption [144]. The final life cycle stage of food is the food waste that most of the time ends in landfills where due to its high biodegradability, it contributes to global GHG generation [145]. Food waste is a waste of resources with environmental implications (resources are needed for food production, transportation, storage, preparation) [146]. However, consumption can be an entry-point for thinking about climate change ethical responsibility. There is evidence that consumers are turning to more environmentally responsible products and services [147]. For instance, local food alternatives will likely play an important role in building a food system resilient to climate change [146]. “Local food” contributes significantly to lower GHG emissions [148]. Additionally, the label is linked to the miles from the production site to the consumer and therefore to a specific carbon footprint or a particular pattern of use pattern [149].

The scientific community acknowledges the uneven distribution of climate change effects. Additionally, research on perceptions has revealed that climate change is most often associated with a belief in higher impacts for developing countries, considered less adaptable to climate change, or for future generations [73,75]. One explanation stands in the slow-moving nature of climate change that makes it difficult for people to directly perceive and experience it [85,150]. Practically, our difficulty in detecting climate change effects amid the normal variation of daily weather is one of the main obstacles to being aware of its existence and taking adaptation measures [68].

Now, to come back to climate change solutions, in the following, we refer to one of the most studied and debated climate change solutions: decarbonization. The disparity between the measures and effects of the decarbonization process is highlighted by Sovacool et al. [82] through the “decarbonization divide” concept. Fossil fuel combustion for energy production causing greenhouse gas emissions (GHG) is an important contributor to climate change [62]. Many studies examined various approaches for reducing GHG emissions, including technological options and managerial strategies per sector [72,81]. The production and energy use account for more than 75% of the EU’s GHG. At the EU level, decarbonizing the EU’s energy system is critical to reaching the 2030 climate objective of GHG reduction by at least 55% compared with 1990 levels and carbon neutrality by 2050, in a context where the production and use of energy represent around 75% of the EU’s GHG [71]. One of the most popular ways to achieve decarbonization is using renewable energy; therefore, many countries already use an increasing share of their renewable resources (e.g., wind, solar, geothermal, or hydropower) [79]. Although most research investigating low-carbon transitions focuses on the use of innovations (e.g., electric vehicles, solar panels, lithium-ion batteries), it overlooks the downstream and upstream processes [82], such as mining, waste flows, biodiversity loss, or change in land use. Taking as a case study Congo and Ghana, Sovacool et al. [82] highlighted how this decarbonization divide is reflected in environmental and public health risks, gender discrimination, child exploitation, and the oppression of ethnic groups. Similarly, other studies bring to the fore the decarbonization divide that leads to socio-ecological destruction [70,74,83]. Therefore, when considering climate change adaptation strategies, the evaluation of the risk of maladaptation should be a priority for decision-makers and stakeholders [77]. This statement introduces the subsequent science-based climate change topic: adaptation and maladaptation.

### 3.4. Adaptation and Maladaptation

The absence of appropriate pro-environmental behavior is widespread in the context of the proliferation of climate change denialism debates [107]. According to Hornsey et al. [92], there is only a slight relationship between climate change beliefs and the extent to which individuals are willing to act in a climate-friendly manner. The main challenge is not how many people believe in the reality of climate change and its anthropogenic origin but how these beliefs are translated into climate adaptation actions [103].

Even if the science of climate change is full of uncertainty [89], there is plenty of evidence about the vulnerability of the affected communities and the role that science has in informing about the climate crisis [93]. Zooming into the climate change literature, there is a tremendous amount of research [94,98,100,102] dedicated to worldwide climate change adaptation and mitigation both in rural and urban areas. Adaptation is seen as “adjustment in natural or human systems in response to actual or expected climatic stimuli or their effects, which moderates harm or exploits beneficial opportunities”; at the same time, mitigation is “an anthropogenic intervention to reduce the sources or enhance the sinks of greenhouse gases” [95]. It follows from these definitions that mitigation reduces all impacts (positive and negative) of climate change, fostering adaptive capacity, whereas adaptation is selective [91]. Furthermore, mitigation has global benefits, while adaptation typically works on the scale of an impacted system, which is primarily local [97]. Since adaptation to climate operates in the context of numerous uncertainties and unknowns [86], practices can become maladaptive if they increase vulnerability [87,88].

Based on a literature review, Juhola et al. [96] distinguish between three types of maladaptive outcomes—rebounding vulnerability, shifting vulnerability, and eroding sustainable development. There is a rich amount of research [90,101,105,106] that has documented cases of climate initiatives that turned to maladaptation by undermining local access to resources, land rights, or community solidarity, or by limiting the choices of future generations. For example, to optimize production efficiency to climate variability, the introduction of new crop species and varieties is often mentioned by farmers as an adaptation measure in the agricultural adaptation literature [52,151,152]. Changing crops may have unexpected outcomes such as higher input of fertilizers, increased risk of pests, and weed infestations [99]. Consequently, as Eriksen et al. [90] posited, there is an urgent need to better understand “local” vulnerabilities to cover global contexts and drivers of vulnerability.

### 3.5. Climate Change: A Source of Conflict

People are increasingly dependent on climate-sensitive forms of natural capital, and the less they rely on economic or social forms of capital, the more at risk they are from climate change [14]. Climate change is reported as one of the new sources of instability and conflict [115], and it is perceived as a “threat multiplier” [110,121], intensifying poverty, social insecurity, injustice, violence, and terrorism [116].

Climate variability impacts environmental and human systems, leading to population displacement due to resource depletion, availability of and access to many critical natural resources, and loss of land and property. Therefore, migration could increase the risk of violence, particularly in the context of neo-Malthusian resource scarcity conflict [109], and could also compromise important determinants of health (e.g., water security, shelter, education, food, and access to labor markets) [153]. If we follow the United Nations [121] projections for climate change, population growth, and consumption, we will need the capacity of two earths to keep up with natural resource consumption by 2030. Considerable uncertainty remains regarding climate change’s role as a driver of migration and conflict [109] because there are voices claiming that disasters also produce an increase in social solidarity [117].

Darfur conflict that started in 2003 and brought costs in human life and population displacement is portrayed as the first modern climate change conflict [108,114]. Hakim (2011) offers a detailed picture of the multitude of factors that operated simultaneously in triggering the conflict. The Darfur Conflict began with small-scale armed disputes between “Arab” nomadic pastoralists and African farmers relying on rain-fed production over access to water and arable land in Darfur during extreme drought [118,120].

Using the Social Conflict in Africa Database and covering around 6000 instances of social conflict in Africa, Hendrix, and Salehyan [112] revealed how rainfall and water resources affected political stability. Based on a systematic literature review from 1986 to 2013, Xu et al. [123] showed that natural disasters related to extreme weather conditions increased the risk of conflicts. Similarly, Theisen et al. [122] reviewed the evidence of the link between the changes in precipitation and temperature, rising sea levels, natural disasters, and interstate conflict. However, this link is highly country-dependent. States with large populations, non-respect of the rights of ethnic minorities, corruption, and a low level of human development are more vulnerable [113].

### 3.6. The Heroes, Villains, and Victims of Climate Change

The narrative, as a particular category of communication, has characters, besides other structural elements (e.g., a setting, a moral of the story). These are the heroes as characters who are fixers of the problem, the villains who are the causes of the problem, and the victims who are those harmed by the problem [128]. The climate change literature testifies [26,124,129,130,132] on the evolving character of climate change storylines.

Han and Ahn [127] addressed young activists’ understanding and responses to climate change. Based on youth discourses, speeches, and websites, the authors introduced the characters of the climate change narrative as heroes, villains, and victims. The victims were the Earth, the ecosystem, younger generations, marginalized groups, and weak states; the heroes referred to the younger generations, climate science, and the reformed states; the villains were the older generations, the media, the fossil fuel industry, and the states. Sovacool [82] looked at the low-carbon transition. Based on academic research, he named 24 vulnerable groups (e.g., non-human species, local communities, and future generations) who suffered from experiencing the political ecology of climate mitigation. Jones [26] argued that, for egalitarians, the villains were the profit-driven corporations, governments, and any group that sustained those corporations. The heroes were the groups such as Ecodefense and Earthfirst that fought for GHG reduction and advocated for fundamental changes in the human–nature relationship. In a study dedicated to climate change lifestyle narratives among Norwegian citizens, the authors [126] showed that Norwegians could not or were unwilling to assign responsibility to specific individuals, groups, or institutions when telling the story of climate change.

Lück et al. [131] approached the victim–villain–hero constellations in a comparative quantitative analysis of the newspaper coverage in five countries. They found two patterns. On the one hand, in German and US newspaper coverage, both the victims and the villains of climate change are less individualized, encompassing developing countries and small island states, and humankind and past generations, respectively. Moreover, often villains are explicitly named (e.g., emerging countries are judged as villains in the US newspaper coverage). On the other side, in South Africa, Brazil, and India, their home countries are portrayed as victims and heroes. At the same time, the image of the villains is also unambiguous—rich and developing countries are responsible for climate change problems.

From this science-based climate change topic that could be interpreted as “Who are the climate-change heroes, villains, and victims?”, we move to the following subsection that introduces the question “Who contributes the most to climate change?”.

#### “The King” of Climate Change Villains

We used a closed question (“What do you consider to be the “king” of climate change villains?”) with seven possible answers for this science-based climate change topic. In the following, we justify the choice for the answer options based on the reviewed literature (in addition to the explanations included in the Methodology section).

The European Environment Agency [25] presents the following sources of human-produced greenhouse gases as the most important ones: (a) burning fossil fuels for electricity production, transportation, industry, and households; (b) changes in agriculture and land use, such as deforestation; (c) waste storage; and (d) the use of fluorinated industrial gases. For the last source, the participants received an additional explanation (“Fluorinated gases have a strong global warming effect, up to 23,000 times greater than CO_2_. They have great applicability in industry, e.g., serving as refrigerants in refrigeration, air conditioning and heat pumps, foaming agents, and additives in the extraction of natural products such as nutraceuticals and flavorings”).

For answer option “Overproduction-consumption nexus,” we referred to Stuart et al.’s [139] study, where they illustrated how production drives consumption and the overconsumption implications for addressing climate change. It was considered that since GDP is an index of production and increasing GDP translates into an increase in energy and material use [139], a positive relationship between GDP and GHG emissions can be found [133].

The answer option “Population growth” was added starting from IPCC’s consideration (cited by [136]) that population growth was one of the leading causes of increased GHG emissions and accelerated global climate change. However, Satterthwaite [138], based on a review of carbon dioxide CO_2_ emissions between 1980 and 2005 (and between 1950 and 1980), showed little association between nations with rapid population growth and countries with high and fast GHG emissions. Studies argued that it was not the population growth that caused the growth in GHG emissions but rather the growth in consumption levels [138]. Therefore, population growth and increasing global consumption of resources nexus are the primary keys in which the overpopulation contribution to climate change is judged [134,135].

## 4. Results

### The Reflection of the Six Science-Based Climate Change Topics in Belgians’ and Romanians’ Minds

Storylines gained increasing attention in studies of climate change [3,12,18,65,80,127,154,155,156] because they communicate and shape opinions and perceptions of risk related to climate change, thus affecting behavior towards adaptation and mitigation. This section answers the second EQ, “How do the investigated people resonate with science-based climate change topics?” and reveals how Belgians and Romanians experience and interpret the six selected science-based climate change topics. Table 2 presents the demographic profile of the participants and Table 3 highlights the commonalities and differences between the Belgians’ and Romanians’ views.

**Figure 1:**
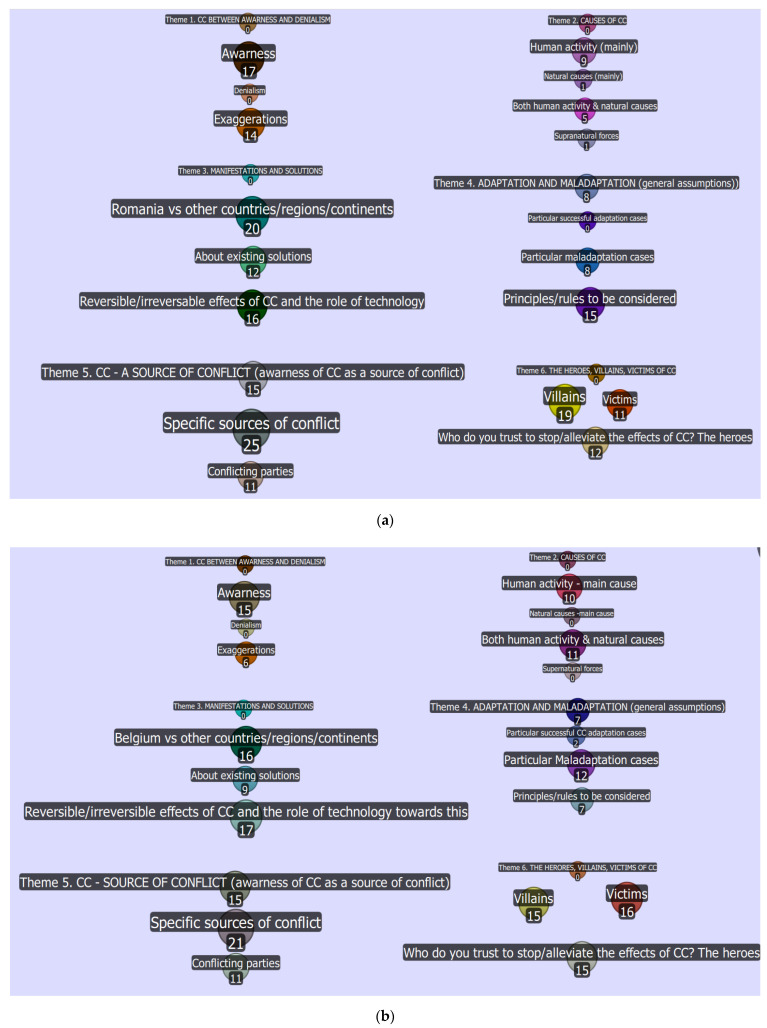
(**a**,**b**). The matrix of the six science-based climate change topics (the “Quirks”) and their subtopics (generated in Quikos software 2.4.1). (**a**) Romanian participants. (**b**) Belgian participants.

The number assigned to each bullet (Figure 1a,b) indicates the number of participants’ statements associated with that subtopic. The higher the number assigned to a subtopic, the bigger the bullet becomes. Quirkos facilitated the visual representation of text.

**Figure 2:**
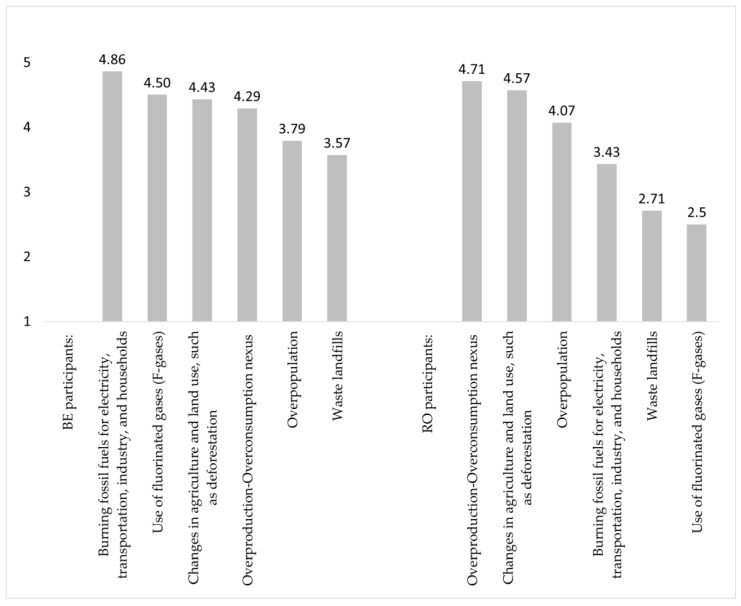
“The King” of CC villains (Belgian and Romanian participants).

For theme 6.1. “The King” of CC villains, Romanians assigned the highest score to “Overproduction-overconsumption” among the main contributors to climate change (Figure 2). In contrast, Belgians placed that contributor the fourth. “Waste landfills” was assigned a similar low score by Belgians and Romanians. Three Romanians participants filled in the open question “Name other sources that contribute to climate change”, and they mentioned “The corruption that leads to wrong actions, such as the deforestation in Romania,” “Supersonics that affect the ozone layer,” and “The wickedness and greed of people who want more and more.” The Belgians added “Natural variations of the climate before the industrial revolution”, “Selfishness and ignorance”, “Transportation”, and “The destruction of oil infrastructures during the Gulf War and volcanic eruptions.”

## 5. Discussion

The thematic analysis revealed that both Romanian and Belgian participants are aware of climate change. Similar, in a qualitative study, Sorgho et al. [157] found that Burkina Faso population at all levels is aware of climatic changes in their environment, which they considered intertwined with the agricultural and economic development of the country.

Even if all Romanians considered that climate change exists, there is a general opinion that it is a politicized and exaggerated issue: *“(…) it is evident that it (climate change) is used as a pretext to favor certain types of production/products marketing or to ban them. Politicians also use it as a national and global manipulation tool.”* (Daniela, Ro), *“(…) out of five scoops on Google, two are about climate change. Climate change is also blamed for the energy prices, justifying that they must act to stop CC in this way, too”* (Doina, Ro). One explanation of these participants’ position could be that climate change communication has taken place through media and social messengers, to the detriment of scientific communication. Areia et al. [158] considered that the communication of climate change is one of the causes of the social inertia causing the awareness–action gap. Whitmarsh [159], who investigated the British public, observed that the proportion agreeing climate change topic was exaggerated doubled over 2003–2008. Recently, using a unique dataset of surveys and interviews with residents of the U.S. Pacific Northwest, Haltinner and Sarathchandra [43] reported for the statement “potentially negative effects of climate change have been exaggerated” a mean of 4.56 (measured on Likert scales from strongly disagree = 1 to strongly agree = 5). Six Belgian participants considered climate change as a pretext for some politicians to impose restrictive measures, but their majority considered relevant and not at all exaggerated to discuss about climate change *“Climate change is not a pretext to impose restrictions. It is not exaggerated to speak about climate change”* (Victor, Be).

Even if most Romanian and Belgian participants consider anthropogenic causes the leading contributor to climate change, a better understanding of our contributions to climate change can activate norms about social responsibility to correct deficiencies caused by human action [160]. This is all more important since several participants attributed climate change effects exclusively to natural conditions and even to supernatural forces. Research suggests that the denial of the anthropogenic contribution to climate change is a significant cause of concern, since some may believe that there is no reason to adapt to change, and this could be translated to the belief that there is no reason to support climate change coping policies [161]. One example is the United States, where around 32% of people do not believe in human-caused climate change [162].

Another observation is that Romanians, mainly, but also several Belgians perceive experiences of severe weather events such as climate change, a reality also exposed by Farrokhiet al. [163] on Iranians. *“I noticed that compared to 30 years ago, when the snow was one meter high all winter and you couldn’t get out of the house, now, it doesn’t exist. It’s snowing one day, and that’s all. Then, the summers are stormy. It used to be so hot that you couldn’t sleep at night in the south.” (Monica, Ro)*, “*For several years in Europe, the temperature has been higher in winter, with less snow and frost. More droughts in southern countries.”* (Chloë, Be). Weber [160] argued that people often falsely attribute unique events to climate change. Thus, they fail to detect climate changes, which is a phenomenon complicated to identify by the public, using their standard tools of observation and inference. It was observed that there were respondents who used temporal anchors when discussing climate change impacts (they tended to push the visible and more severe effects of climate change into the future, the next 30 years, for example). These temporal anchors lower the moral intensity of climate change [164] by increasing the perceived distance between cause and effect and hence the need for immediate actions. Considering these temporal anchors, Gough and Shackley [165] posited that much will depend upon the occurrence of extreme weather events and whether these are perceived as being caused by climate change. In response, it has been suggested that climate action should reveal the immediate consequences of climate change in the here and now [166].

Most of the Romanian participants seemed to be optimistic about nature’s capacity to regenerate, but they linked it to a more environmentally friendly behavior shift: *“Nature can regenerate if we become more restrained”* (Doina, Ro), *“Nature is smart; it can regenerate itself if we are more responsible”* (Monica, Ro). Skeptical voices were not missing either: “*Maybe in some parts, the effects of CC are irreversible, while in other regions, not, as is the case with the Aral Lake, where nothing can be done”* (Maria, Ro). There was also a general view among Romanians that technology can counteract the effects of climate change. Still, its costs and availability could be a barrier: *“Technology will be a solution, but not now, because states are not financially prepared for such a thing, especially since we are talking about changes in the pandemic when the economies are impoverished anyway”* (Horea, Ro). Most of the Belgians did think that technology could be a solution. They were not as optimistic as the Romanians in this respect. *“New technologies could help but are not the solution itself*” (Gabriel, Be). Bonaccorsi et al. [167] warn that people tend to be overly optimistic about technology, and often, the primary responsibility of climate change mitigation is put on technological advancements [168].

Interviewed people voiced principles that should be observed when implementing climate change strategies. These principles range from solidarity, cooperation, and consultation, to a “solid scientific basis when implementing new strategies” or “to rely on people’s expertise rather than economic and political interests,” which ask for a more visible scientific message within the community and less politicization of climate change. Unfortunately, one of the leitmotifs of the interviews with the Romanian participants was corruption and lack of trust in the political class, regardless of the six topics they referred to: *“There is too much corruption, and politicians are just thinking about making money”* (Radu, Ro). This is not surprising since Romania is globally ranked 69th by its perceived levels of public sector corruption (where 1st is the least corrupted and 180th the most corrupted country) [169]. Only one Belgian referred to corruption when it came to those who they trusted to overcome the climate change impacts: *“I do not trust anyone. I think that the world is too corrupted and that every initiative will fail”* (Nora, Be).

All the Romanian interviewees were unaware of successful climate change adaptation measures/strategies, and only two Belgians referred to such successful actions, while they gave many examples of maladaptation. Some Romanian participants keenly pointed out the ban of using wood for household heating, which was widely publicized in Romania in the last months of 2021 (a ban that was finally postponed), as maladaptation since that interdiction was linked to the achievement of the climate neutrality goal. Similarly, many Belgians referred to the Green Deal envisaged transition to green energy (including the case of electric cars) as a maladaptation case. Several Belgians referred to organic products, which they considered a more expensive and natural resource than conventional ones, as a case of climate change maladaptation. This perceived reality asks for media coverage of positive and successful examples of the fight against climate change.

Moreover, educational and informational campaigns are needed to raise awareness of organic agriculture as a positive example of how farmers can help mitigate climate change. This is all more important since the Belgian respondents were from Wallonia, where 90.8% of the country area dedicated to organic farming is located [170]. Opinions did not differ between countries on whether climate change is, will, or could be a source of conflict. They further referred to the lack of food, shelter, or water over which people conflict. One interviewee considered that the lack of decision-making power of small producers regarding why and how to produce could also be a source of conflict triggered by climate change.

For the last science-based topic, politicians were blamed by most Romanians as the main responsible for climate change. However, participants offered a wide range of “villains”: *“The great powers—Russia, the USA, China—and the rich of the world are to blame for what is happening, and Romania’s politicians”* (Iuliana, Ro). It is essential to underline that only three of the Romanian participants accused ordinary people, themselves, of contributing to climate change. Most Belgian interviewees acknowledged that everyone is responsible for climate change. At the same time, most Romanians stated that they were the victims of climate change. Mazutis and Eckardt [164] called this dilution of individual responsibility “It’s Not My Problem” bias that can be translated into a lack of reaction, a passivity of pro-environment behavior. Practically, there is very little space to establish concern about climate change and act without the feeling of responsibility. On the contrary, Belgians pointed towards poor people as the main victims of climate change.

The discussions showed that Romanian participants emphasized general, broad categories of climate change “villains” (e.g., politicians, multinational companies, and big countries). This is similar to Greek university students who named economic growth and industrialization the main culprits of climate change [125]. Only 6 Romanians out of 14 pointed towards specific “villains” of climate change. Thus, China was the most frequently mentioned power contributing to climate change. This could be explained by the considerable attention China received in media for its air pollution, energy consumption, and, in general, for resource consumption [171,172]. Belgians provided no references to politicians, specific states, companies, or persons. Instead, like Romanians, they referred to broad categories responsible for climate change: *“Human activities, our Western cultural heritage centered on infinite production, and politicians”* (Téa, Be).

Finally, we revealed a set of climate change portrayals described by participants based on their similar views related to the six science-based topics. These portrayals must be valued as an inventory of local visions and familiar storylines [18] regarding the six climate change topics. Therefore, we considered that the best way to mirror these portrayals is through the participants’ thoughts (Table 4).

The present study testifies that participants’ economic and political beliefs influence their views on climate change. This creates an environment of rhetorical adversity where misinformation is present [173]. Although the findings are not sufficient to make a conclusive statement on the extrapolation of the views of participants on climate change to the entire population of Romania and Belgium, we stress that this is the first paper dedicated to investigating Romanians’ and Belgians’ perceptions of climate change, which brings to the fore the importance of a cultural reading of climate change. We acknowledge that there are several limitations of the research. A qualitative approach was used to offer a more fine-grained understanding of differences and similarities between two groups on a wide range of topics related to climate change. Therefore, a limitation refers to the lack of representativeness of this qualitative study, since qualitative research methods are not concerned with generalizing to a larger population and does not tend to rely on hypothesis testing but rather are more inductive [174]. Therefore, in quality research, the extent to which the findings apply to situations and people outside and beyond the scope of the investigation remains uncertain [175]. Future thematic analysis could focus on the content of newspapers’ articles that contain the terms “climate change” and “fake news”, and the role of climate change “fake news” in shaping and reshaping perceptions. Next, cultural differences may play a role [176] and also political ideology and party identification are essential factors for resonating with different climate change issues [177]. This aspect alone would require a separate investigation. Probably, other key climate change problems could have been identified to have a more realistic picture of how science-based climate change topics are reflected in people’s minds.

## 6. Conclusions

We documented evidence about a set of science-based climate change topics in the climate literature, and we elicited Romanians and Belgians perceptions about them. It was revealed that perceptions about climate change, its causes, and its impacts are social constructs with a high degree of variability between and within nations (Belgian and Romanian, in the present case). On the one hand, the thematic analysis highlighted many commonalities between the two groups of participants, such as the awareness of climate change, climate change as a source of conflict, and transition to green energy as a source of climate change maladaptation (see Table 3). On the other hand, there are several profound differences between the two groups. Climate change was perceived as an exaggerated topic only by Romanians. Belgians saw science as the solution to climate change, whereas Romanians were skeptical about trusting someone or something to overcome climate change impacts. Practically, climate change portrayals described by participants, extracted from their perception of the six science-based climate change topics, showed that climate change was locally embedded.

The findings provide insights for policymakers and other practitioners who want to connect better the governance of climate adaptation with citizens’ perceptions of climate change. Summing up, the discussion has shown several pathways to be considered in addressing climate change.

Firstly, the identified cleavages between scientific communication and participants’ perceptions are the blind spots in which a participatory approach of scientists, media, politicians, and citizens is needed to better cope with climate change challenges. For example, one intervention is required to adjust Romanians’ perceptions of climate change as an exaggerated subject. Another intervention point is related to the low scores assigned by Romanians and Belgians’ participants to the impact of waste on climate change. More information and education campaigns are needed to overcome the citizens’ knowledge gap. Secondly, both in Romania and Belgium, heightened attention should be placed on the climate change scientific consensus rather than controversies to raise public acceptance. Thirdly, in line with Mauelshagen and Pfeiffer’s [47] conclusion, we consider that climate scientists all over the world should practice political abstinence. Fourthly, we observed that Romanians and Belgians rarely referred to successful climate change adaptation programs while maladaptation situations dominated their answers. This implies a particular focus of the mediatic campaigns towards highlighting the positive outcomes of the EU climate change policies. Consequently, there is also a strong need for media coverage of positive and successful examples of fighting against climate change. Fifthly, successful climate change messages should be tailored to include cultural variation (in values, norms, and institutions). Having most Romanians believing that climate change is an exaggerated topic, a politicized instrument to impose more restrictions, and Belgians voicing the contrary, it is justifiable to place Romanian and Belgian participants on a “skeptics” and “supporters” dichotomic axis.

The authors acknowledge that while the purpose of this research was to qualitatively explore the perceptions of several climate change, the findings are not generalizable on a larger scale. However, this type of qualitative research speaks of the need to investigate perceptions of people from various geographical, political, and economic settings and with different cultural beliefs. Such a qualitative approach could decipher barriers to behavior change in other parts of the world, thus contributing to the common efforts to fight climate change.

## Figures and Tables

**Table 1:** The referenced literature for the scientific-based climate change topics.

The Scientific-Based Climate Change Topics	The Referenced Literature
Climate change between awareness and denialism	[21,34,35,36,37,38,39,40,41,42,43,44,45,45,46,47,48,49,50,51,52,53]
The causes of climate change	[54,55,56,57,58,59,60,61,62,63,64,65,66,67]
Manifestations and solutions of climate change	[68,69,70,71,72,73,74,75,76,77,78,79,80,81,82,83,84,85]
Adaptation and maladaptation	[52,86,87,88,89,90,91,92,93,94,95,96,97,98,99,100,101,102,103,104,105,106,107]
Climate change: a source of conflict	[14,108,109,110,111,112,113,114,115,116,117,118,119,120,121,122,123]
The heroes, villains, and victims of climate change	[26,82,124,125,126,127,128,129,130,131,132]
“The king” of climate change villains	[25,133,134,135,136,137,138,139]

**Table 2:** Summary of demographic profile of Belgian and Romanian interview participants.

	Romanian Participants (*n* = 14)	Belgian Participants (*n* = 15)
Gender (% out of the sample)	50% male	53.34% male
	50% female	46.66% female
Education (completed level)	57% university level	60% university level
	43% 12 years of education	40% 12–14 years of education
Living area	69% urban	53.33% urban
	21% rural	46.67% rural
Average age (years)	41.50	47.00

**Table 3:** Highlights of participants’ main views towards the “Quirks” and their subtopics and the commonalities (in green) and differences (in orange) between the Belgians’ and Romanians’ views.

The “Quirks” and Their Subtopics	Romanian Participants	Belgian Participants
CC * between awareness and denialism		
Awareness	All respondents were aware of CC existence.	All respondents were aware of CC existence.
Denialism		
Exaggeration	Most (10 out 14) believed CC was an exaggerated topic.	Six Belgians considered CC as a pretext for some politicians to impose measures. Only one participant thought CC was an exaggerated topic.
**Causes of CC**		
Human activity (mainly)	Only two persons believed that natural causes are the main contributor to CC.	No Belgian pointed to natural causes as the main contributor to CC.
Nature (mainly)		
Both human activity and nature	Most people considered that human activity was responsible for CC. However, they also mentioned nature as a contributor, but a minor one compared with anthropogenic impact.	Most people considered that human activity and natural causes are responsible for CC.
Supranatural forces	One person named God as the artisan of CC.	Nobody believed in God or other supernatural causes as a contributor to CC.
**Manifestations and solutions of CC**		
Romania/Belgium vs. other countries, regions, continents	All participants considered that CC is visible in Romania, but CC effects are more visible in other parts of the world. They mainly mentioned Africa as the worst-affected continent.	All participants said that CC is present in their region, but CC is more visible in other parts of the world. They mainly mentioned Africa and South-Eastern countries being the worst-affected.
About the existing solutions	Regarding the implemented/future solutions to combat CC, most participants considered that suddenly applied restrictions would impoverish the population. One person believed that these would help to improve the current situation.	Out of the seven answers that touched on the climate change solution topic, five included the idea that the existing solutions can limit but not stop climate change.
Reversible/irreversible effects of CC and the role of technology towards this	The majority thought that the effects of climate change could be reversible under certain circumstances.	Ten Belgians believed that the effects of CC could be reversible.
	The technology could help if used wisely and not at very high costs.	The majority voiced that the new technologies would find solutions to CC.
**CC adaptation and maladaptation**		
Successful CC adaptation cases	Nobody was aware of successful CC adaptation cases.	Only one person could mention a successful CC adaptation initiative.
Maladaptation cases	The ban on wood for heating was often mentioned in the context of maladaptation. Electric cars, GMOs, and blocking fossil resources exploitation were other themes around which some of the participants justified their answers as maladaptation examples.	The transition to green energy (including the case of electric cars) and the abolition of nuclear power plants without having the security of supply was mentioned as a maladaptation case.
Principles/rules to be considered for CC strategies	“Solidarity”, “cooperation”, “consultation”, “common sense”, and “reasonable, rational, and decent welfare” were most often maintained as principles that should guide the implementation of the measures.	No recurrent principle/rule was identified. They range, for example, from cooperation, global thinking, or systemic vision.
**Climate change: a source of conflict**		
Specific sources of conflict	Everybody opined that CC was or could be a source of conflict. Most participants mentioned land, food, and water as sources of conflict.	Everybody opined that CC was or could be a source of conflict. Most Belgians mentioned water, food, air, migration as sources of conflict.
Conflicting parties	When people referred to conflicting parties, most responses mentioned people vs. their states, states vs. states, small manufacturers vs. corporations, ordinary people vs. politicians.	When Belgians referred to conflicting parties, most named the states, local people, and multinational companies.
**The heroes, villains, and victims of climate change**		
The villains	The answers were very diverse regarding the culprits. They ranged from politicians to multinational companies and big states. One participant named mass media and scientific community, another blamed the past generations (starting with the Industrial Period), and another participant added to the list of CC villains “farmers and the mining industry.” However, the majority pointed to politicians.	Five out of fourteen considered that everyone is responsible for CC. Others named Western cultural heritage and industry as the villains. Nobody mentioned specific countries, multinationals, or politicians.
The victims	Most of the interviewed Romanians considered that ordinary people (themselves) were the victims of CC; only three of them mentioned nature as a victim.	Ten participants believed that poor people (from developing countries) were the victims. Others named as victims the farmers, fishermen, and even the consumers.
The heroes	Four people said they do not trust anyone to stop/alleviate the effects of CC.	Three people said they do not trust anyone to stop/alleviate the effects of CC.
	Three mentioned God as a force who could guide the steps towards solutions; others mentioned the politicians if they changed their habits. Other mentioned lay people if they changed their way of living. One participant considered that a revolution is needed to change the organization, production, and consumption system.	Most Belgians pointed to science as capable to offer the solutions to CC. Other respondents named the youth, local non-profit associations, the associative sectors, or the world organizations as forces who could guide the steps towards solutions. Only one person considered the lay people among those who could deliver a solution.

* CC (Climate change).

**Table 4:** The portrayals generated by the citizens for the six-science based topics.

The Six Science-Based Topics	The Portrayals Generated by the Citizens for the Six-Science Based Topics	
	Romanian Participants	Belgian Participants
CC between awareness and denialism	“**CC exists**, but it is often used as an **exaggerated and politicized** subject” (Monica)	“Yes, **there is climate change** (…), and **it is not exaggerated to talk about climate change**” (Téa)
Causes of CC	“**Human activity is the main cause of CC. Natural causes also contribute**, but not as much as humans” (Zoltán)	“**Human activities and natural changes are the causes of climate change**. Human activities **have the most significant impacts** and produce GEG, which will be stocked in the atmosphere for a long time.” (Victor)
Manifestations and solutions of CC	“**Climate change is present in Romania**. We are dealing with violent storms, droughts, and floods. Also, the seasons have changed… The **effects are more visible on other continents such as Africa and Asia**” (Bogdan) “**Nature can regenerate** to a certain level” (Daniela)	“**Climate change is already present in my region**. Less snow during winter, heavy rains in summer 2021. **Climate change is principally seen in other parts of the world**. South-Eastern countries are impacted mainly by climate change due to increased sea levels (Vietnam, Thailand, Bangladesh, Cambodia, Sri Lanka…)” (Anna) “**Nature could regenerate**, but some changes are irreversible” (Gabriel)
CC adaptation and maladaptation	“If the measures are taken at the national level, **are taken overnight**, and **people are not mentally and financially prepared to deal with them, they will be a failure**, and we will witness social movements.” (Dani) “**Cooperation** between people, politicians, and countries would be beneficial” (Doina)	“The proposed solutionswill not stop the effects of climate change, but surely, they will reduce them.” (Liam) “(…) to have a **systemic vision, global thinking, and local actions**” (Téa)
Climate change: a source of conflict	“**Land, food, and water** can be sources of conflict” (Cristina)	“**Water, fish resources,** and **forests** are sources of conflict” (Anna)
The heroes, villains, and victims of climate change	“The culprits are the **big companies** for irresponsibly exploiting resources and pollution; **governments** are not taking the necessary steps to transform the system; and **consumers** who lack involvement and consume excessively.” (Daniela) “**The victims of CC are us, the ordinary people**” (Monica) “**I don’t know who to trust**” (Mariana)	“**We are all responsible** for climate change, but mainly the **capitalist and ultraliberal system** in which we live.” (Liam) “**Poor people** are the **main victims** of climate change” (Noah) “I am confident **in science** to find solutions.” (Adam)

## Data Availability

The data presented in this study are available on request from the first author.

## Supplementary Materials

The following supporting information can be downloaded at: https://www.mdpi.com/article/10.3390/ijerph19074280/s1. File S1: Supplementary Material—How Climate Change Science Is Reflected in People’s Minds A Cross-Country Study on People’s Perceptions of Climate Change.
