# Supplementary material for: How Climate Change Science Is Reflected in People’s Minds. A Cross-Country Study on People’s Perceptions of Climate Change
Source: Int J Environ Res Public Health. 2022 Apr 2;19(7):4280. doi: 10.3390/ijerph19074280 (PMC8998260; doi:10.3390/ijerph19074280)
Supplement: Supplementary file 1 [file ijerph-19-04280-s001.zip › ijerph-1628015-Supplemental File S1.pdf]

International Journal of
Environmental Research and Public Health

MDPI

Supplementary Material—How Climate Change Science Is Reflected in People's Minds. A Cross-Country Study on People's Perceptions of Climate Change

Guide for the interview

Theme 1. Climate change between awareness and denialism

Do you think there is climate change?

Is climate change a topic that must be discussed? How to you think that climate change and its effects are presented – at their real dimension, exaggerated, or downplayed?

Theme 2. Causes of climate change

a) Which are the causes of climate change? Think about the causes of climate change: do you think that human activity is responsible for these changes? Why? Please say to what extent human activity contributes to climate change (e.g., partial, total).

b) Do natural causes also play a role in climate change?

c) Do you think that other forces cause climate change? Does God or a supernatural power or energy also contribute to these changes?

Theme 3. Climate change Manifestations and solutions

a) Do you think that climate change is already present in your region? Do you believe climate change is visible the same/ more/ or less in other parts of the world compared your country/region? Especially on which continents/countries/regions do you consider climate changes impacts are present?

b) Do you think that the presented solutions (for example, limiting and then banning the use of fossil fuels - oil, natural gas, and coal) are efficient to stop/to reduce the effects of climate change? Do you think that technology will find the necessary solutions to counteract the effects of climate change? Can nature regenerate to cope with climate change? Do you think the impacts of climate change are reversible or irreversible?

Theme 4. Adaptation and maladaptation

Adapting to climate change is necessary to ensure that the impacts will not overwhelm societies and ecosystems around the world. But often many adaptation strategies fail. This is called maladaptation. Maladaptation is a process through which people become even more likely to be negatively affected by climate change. For example, if people are relocated from coastal areas to avoid being flooded or affected by storm surges, these hazards will no longer pose a threat; however, if they are fishers their coastal access may be constrained, resulting in the inability to keep a protective eye on their boats and fishing equipment, with adverse impacts on incomes and livelihood security.

a) Have you heard about negative consequences of actions/strategies/plans/programs that aimed for climate change adaptation? Do you have any example in mind?

b) If you think about the effects of climate change plans, programs implemented at the national level (in your country), do you consider that their effects are more towards adaptation or maladaptation? Can you develop your answer?

c) Can you think of any principle, rule, strategy that should be followed, applied to prevent maladaptation?

Theme 5. Climate change – a source of conflict

There are documents and studies that consider that climate change leads to depletion of natural resource (e.g., water, land, forests) in many regions and, perhaps, globally. The

resulting scarcity of water, food, and livelihoods could lead to increasingly desperate populations that challenge governments, enhancing the risk of intra- and interstate conflict (water conflicts, land use conflicts, food conflicts, sanitary conflicts, etc.).

a) Do you think that climate change can be a source of conflict? Please provide arguments to support your answer. (Yes, because…; No, because….)

b) What resources can be a source of conflict in relation to climate change? Who are the parties in conflict?

c) Do you know concrete examples of such conflicts generated by climate change?

Theme 6. The heroes, villains, and victims of climate change

To give the following answers, please think of a wide range of victims / villains / heroes of climate change.

a) Who do you think are the victims of climate change? Who suffers from climate change?

b) Who do you think is to blame for climate change?

c) Who do you trust can stop/alleviate the effects of climate change?

Theme 6.1 "The king" of climate change villains

Please assign a number from 1-5 to the following sources considering their contribution to climate change (where 1 = very small contribution, 2 = small contribution, 3 = average contribution, 4 = large contribution, 5 = very high contribution). A number can be used for more than one source.

a) Burning fossil fuels for electricity production, transportation, industry, and households

b) Changes in agriculture and land use, such as deforestation

c) Waste storage

d) Use of fluorinated industrial gases (they have high applicability in the industry: e.g., as refrigerants in refrigeration, air conditioning, and heat pumps, as foaming agents, in the extraction of natural products such as nutraceuticals and flavorings)

e) Overproduction-overconsumption, in general

f) Population growth

g) Would you add other sources than those listed above? If so, who and why?

Gender:

Living environment:

Age:

Country:

Education:
